# New *in silico* approach to assessing RNA secondary structures with non-canonical base pairs

**DOI:** 10.1186/s12859-015-0718-6

**Published:** 2015-09-02

**Authors:** Agnieszka Rybarczyk, Natalia Szostak, Maciej Antczak, Tomasz Zok, Mariusz Popenda, Ryszard Adamiak, Jacek Blazewicz, Marta Szachniuk

**Affiliations:** Institute of Computing Science, Poznan University of Technology, Piotrowo 2, 60-965 Poznan, Poland; Institute of Bioorganic Chemistry, Polish Academy of Sciences, Noskowskiego 12/14, 61-704 Poznan, Poland; European Center for Bioinformatics and Genomics, Poznan University of Technology, Piotrowo 2, 60-965 Poznan, Poland

**Keywords:** RNA, Secondary structure, Non-canonical base pairs, RNApdbee, RNAComposer

## Abstract

**Background:**

The function of RNA is strongly dependent on its structure, so an appropriate recognition of this structure, on every level of organization, is of great importance. One particular concern is the assessment of base-base interactions, described as the secondary structure, the knowledge of which greatly facilitates an interpretation of RNA function and allows for structure analysis on the tertiary level. The RNA secondary structure can be predicted from a sequence using *in silico* methods often adjusted with experimental data, or assessed from 3D structure atom coordinates. Computational approaches typically consider only canonical, Watson-Crick and wobble base pairs. Handling of non-canonical interactions, important for a full description of RNA structure, is still very difficult.

**Results:**

We introduce our novel approach to assessing an extended RNA secondary structure, which characterizes both canonical and non-canonical base pairs, along with their type classification. It is based on predicting the RNA 3D structure from a user-provided sequence or a secondary structure that only describes canonical base pairs, and then deriving the extended secondary structure from atom coordinates. In our example implementation, this was achieved by integrating the functionality of two fully automated, high fidelity methods in a computational pipeline: RNAComposer for the 3D RNA structure prediction and RNApdbee for base-pair annotation.

**Conclusions:**

The presented methodology ties together existing applications for RNA 3D structure prediction and base-pair annotation. The example performance, applying RNAComposer and RNApdbee, reveals better accuracy in non-canonical base pair assessment than the compared methods that directly predict RNA secondary structure.

**Electronic supplementary material:**

The online version of this article (doi:10.1186/s12859-015-0718-6) contains supplementary material, which is available to authorized users.

## Background

RNA molecules play an important role in many cellular processes, not only serving as the carriers of genetic information but participating in the regulation of gene expression and acting as catalysts in many biological pathways [1]. These functions result from the sequence and the three-dimensional (3D) shape assumed by the molecule [2]. Thus, any investigation into RNA-involving processes usually requires the study of structural features.

The primary sequence of RNA defines its secondary structure, which in turn designates the 3D fold of the molecule [3]. An analysis of the secondary structure is a crucial step in functional characterization of RNA and its tertiary structure prediction. A classical approximation of the secondary structure considers Watson-Crick AU and GC base pairs, as well as wobble pairs. These three types, regarded as canonical, are the stabilizing factors in the RNA folding process. However, a deep investigation into RNA tertiary interactions, made possible due to the growing number of known RNA 3D structures, revealed a great diversity of other base-base interactions. They are referred to as non-canonical and often defined as neither Watson-Crick (non W-C) nor wobble (not GU or UG). It has been discovered that about 40 % of all bases in structured RNAs take part in non-canonical interactions [4]. Moreover, the secondary structure containing only canonical base pairs proved insufficient for a correct determination of the RNA’s 3D structure and for aligning homologous sequences [5]. Thus, obtaining and describing an extended secondary structure that determines both canonical and non-canonical base pairs is an important issue in RNA structure study.

Various conventions can be applied to describe canonical and non-canonical RNA interactions. One of the first proposals was Saenger nomenclature [3], which distinguished 28 different base-pair classes by their symmetry, base types, and optimization of hydrogen bonding rather than geometry [4]. The other approach, most often used, is Leontis and Westhof’s classification that takes into account the base edges involved in the interaction (Watson-Crick, Hoogsteen or sugar edge), and the orientation of the glycosidic bond with respect to the hydrogen bond (cis and trans) [4, 6]. This approach gave rise to a definition of 12 basic geometric base-pair families that have been observed in experimentally solved crystal RNA structures [4]. It then led to the development of the graphical convention for displaying non-Watson-Crick interactions within secondary structure diagrams, commonly referred to as Leontis-Westhof (LW) representation [4].

*In silico* methods to obtain RNA secondary structure apply either sequence-based prediction or 3D structure-based assessment routines. To our knowledge, over 50 methods have been developed in the former category. Among them, only three can predict the secondary structure in the extended form: MC-Fold [5], MC-Fold-DP [7] and RNAwolf [7]. MC-Fold [5] is part of the pipeline dedicated to *de novo* prediction of RNA tertiary structure. Due to the exponential computational complexity, it is useful for processing sequences only up to 100 nucleotides (nts). This limit is overcome by the MC-Fold-DP version [7] that applies a dynamic programming algorithm. Finally, RNAwolf [7] adopts an enhanced Nussinov algorithm to predict extended RNA secondary structures. As far as non-canonical base pairs are concerned, only MC-Fold provides their complete classification, consistent with LW nomenclature. MC-Fold-DP, while predicting both canonical and non-canonical base pairs, does not distinguish between them in the output. RNAwolf offers the general LW classification of interactions but does not inform about base-pair assignment to a particular isosteric subset within a given geometric family. A common disadvantage of the described applications is that they allow us to predict none (MC-Fold, MC-Fold-DP) or only a small fraction (RNAwolf) of the multi-pairings (eg base triplets) frequently found in RNA structural motifs.

Access to information about non-canonical interactions and multi-pairings is easier when the secondary structure is derived from atom coordinates. RNAView [8], MC-Annotate [9], 3DNA/DSSR [10] and our recently published RNApdbee [11] are tools used for identifying and classifying RNA base pairs, on structural data encoded in PDB files. All of these programs provide base-pair classification according to LW nomenclature and can detect triplets and higher-order base associations. RNApdbee also supports Saenger notation.

Here, we introduce a novel approach to assess the extended RNA secondary structure. The idea is based on predicting the tertiary structure from a user-provided sequence or a secondary structure containing canonical base pairs only, and then back-calculating the extended secondary structure from atom coordinate data. The approach is generic and any method can be used to predict the 3D structure of RNA (eg FARFAR [12], DMD [13], Vfold [14], MC-Fold [5], 3dRNA [15], RNAComposer [16]) or to back-calculate its secondary structure (eg RNAView [8], MC-Annotate [9], 3DNA/DSSR [10], RNApdbee [11]). However, in the case of this application, selecting a fully automated and fast method for 3D structure prediction is preferred, for user convenience. In our proposal, we implement the idea by integrating RNAComposer [16] and RNApdbee [11] functionality in a computational pipeline. RNAComposer, designed as a fast and efficient modeling tool, is employed for automated, high-quality 3D structure prediction of RNA from either a sequence or secondary structure. RNApdbee is used to extract and describe RNA secondary structure from atom coordinate data, taking into account canonical and non-canonical base pairs and multi-pairings. The proposed pipeline supports two usage scenarios. The first and basic one is the prediction of the extended RNA secondary structure from the primary sequence. The second is the extension of an input secondary structure containing canonical base pairs only, by adding information about non-canonical interactions.

We demonstrate that our approach is characterized by computational efficiency, ability to predict and classify a variety of non-canonical base pairs, and the capacity to process RNAs with pseudoknots as well as those with longer sequences, up to 500 nts. It provides easy access to detailed information about canonical and non-canonical interactions that define extended secondary structure of RNA.

## Methods

### From RNA sequence to tertiary structure

There are several methods for homology or *de novo* prediction of RNA tertiary structure from a sequence and/or a secondary structure. For the purpose of our research we have selected RNAComposer [16] — a web server tool for fast, fully automated, high-throughput modeling of large RNA 3D structures. It operates on the RNA FRABASE database [17], acting as a dictionary that relates the RNA secondary and tertiary structure elements. The output RNA model is composed by assembling the 3D fragments, which carry the knowledge about canonical and non-canonical interactions, and the secondary structure topology. RNAComposer works in two modes — interactive and batch — that generally differ in the number of possible input sequences and output models, as well as in the modeling process description details. It allows the user to input a single sequence or secondary structure (interactive mode), or a set of secondary structures (batch mode). If just the sequence is provided, the canonical secondary structure is predicted by RNAstructure [18] (default), RNAfold [19] or CONTRAfold [20] (on user selection). All these tools have been incorporated into the RNAComposer system. Once the RNA secondary structure is available, an *in silico* synthesis of the molecule is completed by composing its 3D model (up to 10 models can be generated for a sequence).

In the RC/Rp pipeline and sequence-based prediction, we decided to use RNAComposer with the default settings: for each input sequence the secondary structure was predicted by RNAstructure and a single output model was generated. The secondary structure-based prediction was run without providing any additional input.

### Extended RNA secondary structure retrieval from atom coordinates

In our proposed pipeline, a computational routine to retrieve an extended secondary structure of RNA from a user-provided PDB file is driven by RNApdbee webserver [11]. At the input, RNApdbee accepts RNA atom coordinate data encoded in a PDB file. Next, it identifies base pairs using incorporated procedures of standalone versions of RNAView [8], MC-Annotate [9] or 3DNA/DSSR [10], on user selection. Additional functions drive classification of non-canonical base pairs according to LW [4, 6] and Saenger [3] nomenclatures, and identify pseudoknot orders. The resulting secondary structure is presented in dot-bracket, BPSEQ and CT formats together with a graphical image. By default, RNApdbee output representations contain only canonical base pairs, while non-canonical ones are included in a separate list. However, the user can also choose to obtain an extended secondary structure with non-canonical base pairs represented in the textual and graphical output. This is a new feature of RNApdbee, not implemented in the first version of the tool. Its selection results in adding non-canonical base pairs to output representations of the secondary structure, providing their classification in a CT file and a separate list supplementing structure description.

### Accuracy measures for RNA secondary structure models

For the purpose of RC/Rp evaluation, the accuracy of predicted extended secondary structures was assessed by computing the number and percentage of predicted non-canonical interactions, precision (PPV), sensitivity (TPR) and the Matthews correlation coefficient (MCC) [21]. Precision, also called positive predictive value (PPV), is the fraction of predicted base pairs that are relevant. It shows the probability of the predicted interaction presence in the reference structure. Sensitivity, also known as recall or true positive rate (TPR), indicates the fraction of relevant base pairs that are predicted as such. Thus, it gives the probability of anticipating base pairs that occur in the reference structure. Finally, the Matthews correlation coefficient (MCC) is the balanced measure of binary classification quality. All of these measures compare a predicted structure with the reference one. Thus, for the purpose of accuracy assessment, the sequences and secondary structures of reference RNAs were collected from the RNA STRAND database [22].

To evaluate predicted models in a large-scale experiment, an automated comparison of predicted and reference non-canonical base pairs was carried out, based on sequences and secondary structures encoded in dot-bracket notation. Since this notation does not support the representation of multiplets, they were not considered. In the case of RNApdbee-annotated models, their dot-bracket representation encodes base pairs connected by more than one H-bond. Consequently, not all interactions occurring in the reference structures could be compared this way.

For a detailed inspection of our pipeline performance, two carefully selected structures — archaeal tyrosyl-tRNA [23] and K-turn linked with GNRA loop — were manually analyzed. The comparison of their predicted models with the reference structures was done on the lists of H-bond connected bases given by particular methods. All other interactions, including stacking, base-sugar, base-phosphate etc., were ignored. A detailed manual analysis, involving PPV, TPR and MCC computation, followed four different variants. In the first, all relevant H-bond mediated base-base interactions (canonical and non-canonical) annotated in the resulting structure were counted as true positives, regardless of their classification. In the next two variants, all relevant canonical (variant II) or non-canonical (variant III) base pairs were taken into account, regardless of their classification. In variant IV, all base pairs that were relevant and correctly assigned to LW categories were regarded as true positives, whereas those incorrectly classified were counted as false positives. In the latter case, the number of false negatives was equal to the number of unpredicted non-canonical interactions.

## Results and discussion

### Computational pipeline to assess extended RNA secondary structure

**Fig. 1:**
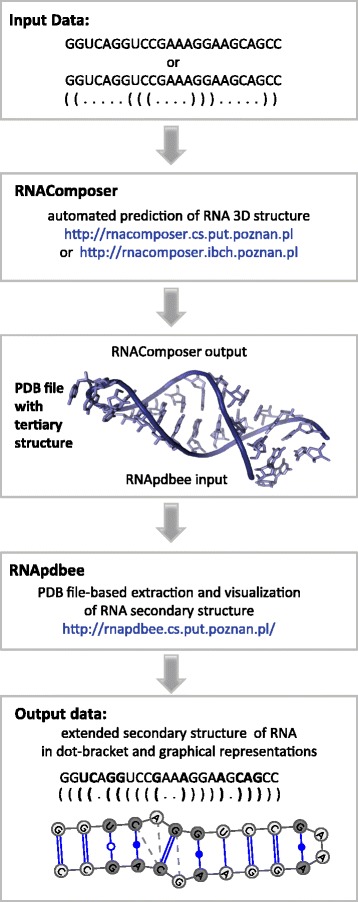
Workflow in the RC/Rp pipeline

The presented method to assess RNA extended secondary structure starts from sequence- or canonical secondary structure-based prediction of the RNA tertiary model and then performs the extended secondary structure retrieval from atom coordinate data. In our proposal, the method is applied by running in a pipeline (the RC/Rp pipeline) two independent web-interfaced applications, RNAComposer and RNApdbee (Fig. 1). In the first step, a user should run a session of RNAComposer, available at http://rnacomposer.cs.put.poznan.pl or http://rnacomposer.ibch.poznan.pl. This application predicts the RNA tertiary structure based on an input sequence of nucleotides or, optionally, a secondary structure. The output model is saved in a PDB file and constitutes the input for RNApdbee, which should be executed in the second step. RNApdbee, hosted at http://rnapdbee.cs.put.poznan.pl/, aims to extract the RNA secondary structure from the PDB-encoded atom coordinate data. It should be run with the *Include non-canonical* interactions option that has been added to the application within the scope of the presented work.

The output secondary model is described by canonical and non-canonical base pairs and referred to as extended representation, in contrast to a non-extended secondary structure that shows canonical interactions only. The resulting structure is encoded in textual notations and displayed in graphical form, both revealing canonical and non-canonical base pairs. Additionally, base-pair classification according to LW and Saenger nomenclature is produced. The results also contain information about other types of interactions such as stacking and interactions formed between sugars, phosphates and bases.

Both components of the RC/Rp pipeline are web server tools available free of charge, designed to work with most common web browsers (Microsoft Internet Explorer, Mozilla Firefox, Opera and Google Chrome). They are fully automatic and do not require any additional information, like templates or sequence alignment, to complete the assessment process. The computation is fast and the results are available immediately.

### Pipeline evaluation and comparison to other methods

To perform a large-scale evaluation of the proposed RC/Rp pipeline and compare it with the other available tools, we have used the data deposited in RNA STRAND [22], a curated database of known RNA secondary structures found in various organisms. Currently, RNA STRAND holds 4666 RNA secondary structures.

For the purpose of evaluation, we decided to retrieve all RNA nucleotide sequences up to 500 nucleotides (nts) long and their associated secondary structures deposited in the RNA STRAND database. Both structures derived from comparative sequence analysis and from tertiary structure determination were included. Next, the data were analyzed and all sequences containing modified residues as well as those with canonical interactions only were excluded. The collection of remaining sequences and secondary structures of 1088 RNAs was divided into four subsets of different lengths (see Table 1): up to 50 nts (319 sequences), 51 to 100 nts (126 sequences), 101 to 200 nts (188 sequences), 201 to 500 nts (455 sequences).

**Table 1:** Quality of non-canonical base pair prediction for RNA STRAND-deposited structures (best values in bold)

		Sequence length (nts)			
		1–50	51–100	101–200	201–500
Number of reference structures that include non-canonical base pairs		319	126	188	455
Total number of non-canonical base pairs observed in reference structures		641	300	607	2252
(a) Results for non-canonical base pairs predicted from sequence					
Number (and percentage) of correctly predicted non-canonical base pairs present in the reference structures	RNAwolf	171 (26.68)	38 (12.67)	44 (7.25)	149 (6.62)
	MC-Fold-DP	**405 (63.18)**	94 (31.33)	157 (25.86)	636 (28.24)
	MC-Fold	363 (56.63)	82 (27.33)	167 (27.51)	n/a
	RC/Rp-1	369 (57.57)	**111 (37.00)**	**291 (47.94)**	**690 (30.64)**
	RC/Rp-2	311 (48.52)	79 (26.33)	244 (40.20)	618 (27.44)
	RC/Rp-3	312 (48.67)	81 (27.00)	225 (37.07)	654 (29.04)
Total number of predicted non-canonical base pairs	RNAwolf	893	501	1334	8616
	MC-Fold-DP	1099	1040	2891	20123
	MC-Fold	816	699	1825	n/a
	RC/Rp-1	1493	1462	4453	26050
	RC/Rp-2	1418	1235	4041	27282
	RC/Rp-3	949	698	2968	14756
(b) Results for non-canonical base pairs predicted from canonical secondary structure					
Number (and percentage) of correctly predicted non-canonical base pairs present in the reference structures	RNAwolf	214 (33.39)	67 (22.33)	268 (44.15)	772 (34.28)
	MC-Fold-DP	n/a	n/a	n/a	n/a
	MC-Fold	334 (52.11)	136 (45.33)	279 (45.96)	n/a
	RC/Rp-1	**452 (70.51)**	**173 (57.67)**	**337 (55.52)**	**1124 (49.91)**
	RC/Rp-2	398 (62.09)	131 (43.67)	290 (47.78)	974 (43.25)
	RC/Rp-3	408 (63.65)	145 (48.33)	261 (43.00)	1051 (46.67)
Total number of predicted non-canonical base pairs	RNAwolf	352	154	461	2183
	MC-Fold-DP	n/a	n/a	n/a	n/a
	MC-Fold	335	137	279	n/a
	RC/Rp-1	1404	1470	4145	26479
	RC/Rp-2	1273	1191	3978	26287
	RC/Rp-3	969	672	2754	15011

All datasets were used to compare the quality of predictions obtained from RNAwolf, MC-Fold, MC-Fold-DP and RC/Rp pipeline in two experiments: the first concerning sequence-based prediction and the second, canonical secondary structure-based prediction. RC/Rp was executed with each option for base-pair identification, namely RNAView, MC-Annotate and 3DNA/DSSR (hereinafter RC/Rp-1, RC/Rp-2, RC/Rp-3, respectively). In the sequence-based experiment, every considered sequence was an input to each of the above-mentioned methods that was executed to predict the corresponding secondary structure. In the other experiment, we applied all the methods except for MC-Fold-DP, which does not accept secondary structure as input data. In case of the dataset containing long sequences (over 200 nts long), MC-Fold computation was interrupted after seven days (during this time the tool managed to process 14 out of 455 sequences). Thus, starting from the set of 1088 sequences, we obtained 11058 secondary structures for further analysis (including 6073 structures in the sequence-based experiment and 4985 structures in the canonical secondary structure-based experiment). Each predicted extended secondary structure was compared to the reference one, retrieved from the RNA STRAND database. On this comparison, the number and percentage of predicted reference non-canonical base pairs was computed (Table 1), taking into account all base pairs that could be encoded in dot-bracket notation.

Since some structures deposited in the RNA STRAND database have been annotated making use of RNAView (applied also in RC/Rp-1), we have run additional experiment to check whether there is an effect in using the same tool for reference and predicted structure. In this experiment, we have selected a subset of all experimentally determined structures deposited in RNA STRAND and we have downloaded their atom coordinates from Protein Data Bank. Every PDB file has been processed separately by RNAView, MC-Annotate and 3DNA/DSSR, which resulted in obtaining three versions of every reference secondary structure. Next, we have run RC/Rp-1, RC/Rp-2 and RC/Rp-3 for each sequence in the subset to compare the resulting secondary model with three versions of the corresponding reference structure (Additional file 1: Table S1). We have also compared every version of the reference structure with respective secondary models predicted from canonical secondary structure (Additional file 1: Table S2). The results obtained for different versions of the pipeline differ only by 0.01–0.04 which proves that the effect of using the same tool is negligible.

Due to the fact that non-canonical base pairs are underrepresented in RNA STRAND-deposited structures (statistically, for each structure in this database, only 2–3 % base pairs are non-canonical), we have computed the total number of such interactions predicted for every structure by the considered methods. In case of structures provided by the RC/Rp pipeline, most of predicted non-canonical interactions can be treated as reliable, since they are derived from atom coordinate data. Thus, even if they are not present in the reference structure, they can be regarded as true positives (Table 1). An additional experiment performed to potentially distinguish between true and false positives has been performed on the benchmark set available from CompaRNA website [24]. We have compared predicted models to reference structures, which were derived for RNAs with experimentally determined atom coordinates (Additional file 1: Table S3). By computing precision, sensitivity and Matthews coefficient, we have evaluated non-canonical base pair prediction from canonical secondary structure as well as canonical and non-canonical base pair prediction from sequence. Due to canonical base pair involvement, we have run the experiments also for RNAfold [19] and CONTRAfold [20] - two methods for canonical RNA secondary structure prediction that are incorporated into the RNAComposer system. CONTRAfold performs very well in predicting canonical interactions which can be observed based on TPR and MCC values. Yet, RC/Rp-1 and RC/Rp-3 are not far behind, moreover, they turn out to be the best if non-canonical base pair prediction is concerned.

**Fig. 2:**
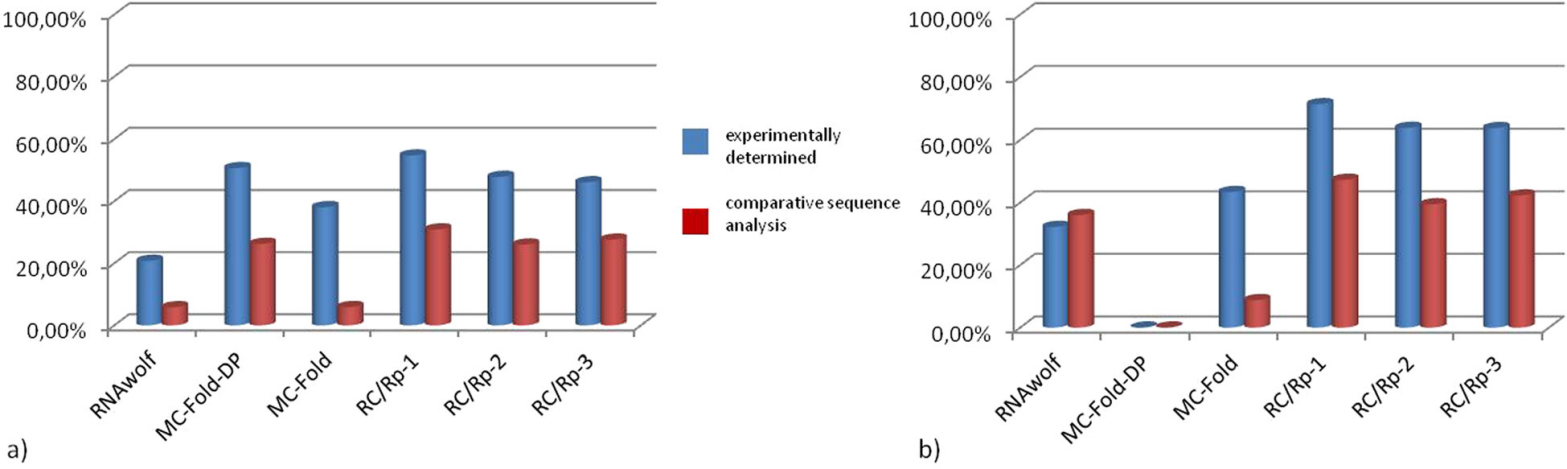
Percentage of reference non-canonical base pairs predicted from (**a**) sequence and (**b**) canonical secondary structure

Based on the input data specificity, we have split experimental results summarized in Table 1 into two parts (Fig. 2). In both the set of input sequences and the set of input canonical secondary structures, we have distinguished data acquired on comparative sequence analysis (668 structures with a global number of 2591 non-canonical base pairs) and structures determined experimentally (420 structures including 1209 non-canonical base pairs in total).

The quality of models predicted from short sequences can be considered reasonably good for all tested methods, with best results achieved by RC/Rp-1 and MC-Fold-DP. For longer sequences, the differences between the methods become more evident. In particular, RC/Rp-1 outperforms the other tools and shows the biggest hit ratio for most subsets. The differences in structures predicted by three variants of RC/Rp result from the varying performance of procedures that identify and classify RNA base pairs. In general, these methods present a broad consensus as to the location of canonical base pairs and stacking interactions, but they are not always consistent when it comes to non-canonical pairs and other types of interactions. MC-Annotate is considered more strict than RNAView, while 3DNA/DSSR takes into account non-canonical base pairs located in helical regions only [11]. MC-Fold-DP applies a knowledge-based potential derived from analyzing the database of 3D structures and performs better than MC-Fold and RNAwolf, but does not distinguish between canonical and non-canonical interactions in the output. Thus, an identification of various types of base pairs must be done by the user in additional sequence-based analysis. We found that in general, predictions for non-canonical base pairs are better for reference structures that have their atom coordinate data determined in an experimental manner (Fig**.**2). Interestingly, this is true for all the methods, even those that predict an extended secondary structure directly from the sequence. However, finding the reason for such input data influence on prediction accuracy requires more detailed investigation, which cannot be done automatically in a large-scale experiment.

Separately, computing times were collected for every method (Table 2). Obviously, due to the difference between our approach and methods that directly predict secondary structure of RNA, computing times of the RC/Rp pipeline are longer than those of RNAwolf and MC-Fold-DP. In the case of RC/Rp, most of the time is occupied by the first step, in which the tertiary structure is predicted. However, the RC/Rp pipeline is still faster than MC-Fold, and obtaining high-quality results should be well worth a longer wait.

**Table 2:** Average computing times (and standard deviation) for RNA STRAND-deposited structures (in seconds)

Method	Sequence length (nts)			
	1–50	51–100	101–200	201–500
(a) Results for sequence-based prediction				
RNAwolf	9.51 (0.25)	9.80 (0.26)	10.61 (0.61)	37.44 (19.15)
MC-Fold-DP	1.62 (0.38)	1.67 (0.48)	1.87 (0.48)	6.63 (2.03)
MC-Fold	6.50 (5.82)	142.26 (124.27)	1376.01 (992.24)	n/a
RC/Rp-1	12.15 (2.78)	20.94 (4.88)	33.38 (4.74)	92.81 (23.39)
RC/Rp-2	12.22 (2.82)	21.20 (4.89)	34.00 (4.81)	97.27 (24.41)
RC/Rp-3	12.17 (2.81)	20.99 (4.90)	33.56 (4.73)	93.40 (23.53)
(b) Results for sequence and canonical secondary structure-base prediction				
RNAwolf	5.71 (4.05)	3.38 (3.93)	5.71 (4.05)	15.06 (58.73)
MC-Fold-DP	n/a	n/a	n/a	n/a
MC-Fold	1.87 (2.83)	35.72 (71.92)	825.68 (1033.23)	n/a
RC/Rp-1	10.64 (3.30)	17.47 (4.19)	29.44 (4.19)	83.97 (23.05)
RC/Rp-2	10.72 (3.35)	17.73 (4.23)	29.99 (4.21)	86.58 (24.03)
RC/Rp-3	10.67 (3.34)	17.53 (4.22)	29.62 (4.21)	84.44 (23.13)

### Application examples

Here we present how the RC/Rp pipeline (ie RC/Rp-1, RC/Rp-2, RC/Rp-3), RNAwolf, MC-Fold and MC-Fold-DP predicted the extended secondary structure of two example molecules, archaeal tyrosyl-tRNA and K-turn–GNRA construct. For both examples, the secondary models were predicted from sequences and compared to the reference structures. By this comparison, including canonical and non-canonical interactions, the PPV, TPR and MCC values were calculated (see Tables 3 and 4).

**Table 3:** The accuracy of secondary structure models predicted from the sequence of K-turn–GNRA construct (best values in bold)

Method	PPV	TPR	MCC
Variant I: Canonical and non-canonical base pairs			
RNAwolf	0.67	0.44	0.54
MC-Fold-DP	0.85	0.61	0.72
MC-Fold	0.77	0.56	0.65
RC/Rp-1	**1.00**	0.67	0.82
RC/Rp-2	**1.00**	**0.72**	**0.85**
RC/Rp-3	**1.00**	0.67	0.82
Variant II: Canonical base pairs only			
RNAwolf	0.70	0.78	0.74
MC-Fold-DP	0.69	**1.00**	0.83
MC-Fold	0.89	0.89	0.89
RC/Rp-1	**1.00**	**1.00**	**1.00**
RC/Rp-2	**1.00**	**1.00**	**1.00**
RC/Rp-3	**1.00**	0.89	0.94
Variant III: Non-canonical base pairs only, regardless of classification			
RNAwolf	0.50	0.11	0.24
MC-Fold-DP	n/a	0	n/a
MC-Fold	**1.00**	0.11	0.33
RC/Rp-1	**1.00**	0.33	0.58
RC/Rp-2	**1.00**	**0.44**	**0.67**
RC/Rp-3	0.75	0.33	0.50
Variant IV: Non-canonical base pairs only, classification dependent			
RNAwolf	**1.00**	0.11	0.33
MC-Fold-DP	n/a	n/a	n/a
MC-Fold	**1.00**	0.11	0.33
RC/Rp-1	0.67	0.25	0.41
RC/Rp-2	0.75	**0.38**	**0.53**
RC/Rp-3	0.67	0.25	0.41

**Table 4:** The accuracy of secondary structure models predicted from tyrosyl-tRNA sequence (best values in bold)

Method	PPV	TPR	MCC
Variant I: Canonical and non-canonical base pairs			
RNAwolf	0.71	0.44	0.56
MC-Fold-DP	0.41	0.33	0.37
MC-Fold	0.57	0.44	0.50
RC/Rp-1	0.94	0.74	0.83
RC/Rp-2	**0.97**	**0.77**	**0.86**
RC/Rp-3	0.94	**0.77**	0.85
Variant II: Canonical base pairs only			
RNAwolf	0.80	0.76	0.78
MC-Fold-DP	0.28	0.43	0.35
MC-Fold	0.56	0.71	0.63
RC/Rp-1	0.95	**1.00**	0.98
RC/Rp-2	**1.00**	**1.00**	**1.00**
RC/Rp-3	**1.00**	**1.00**	**1.00**
Variant III: Non-canonical base pairs only, regardless of classification			
RNAwolf	0.25	0.06	0.12
MC-Fold-DP	n/a	0	n/a
MC-Fold	0.67	0.11	0.27
RC/Rp-1	0.89	0.44	0.63
RC/Rp-2	**0.90**	**0.50**	**0.67**
RC/Rp-3	0.82	**0.50**	0.64
Variant IV: Non-canonical base pairs only, classification dependent			
RNAwolf	0.25	0.06	0.12
MC-Fold-DP	n/a	0	n/a
MC-Fold	0.33	0.06	0.14
RC/Rp-1	0.78	0.39	0.55
RC/Rp-2	**0.80**	**0.44**	**0.60**
RC/Rp-3	0.55	0.33	0.43

**Fig. 3:**
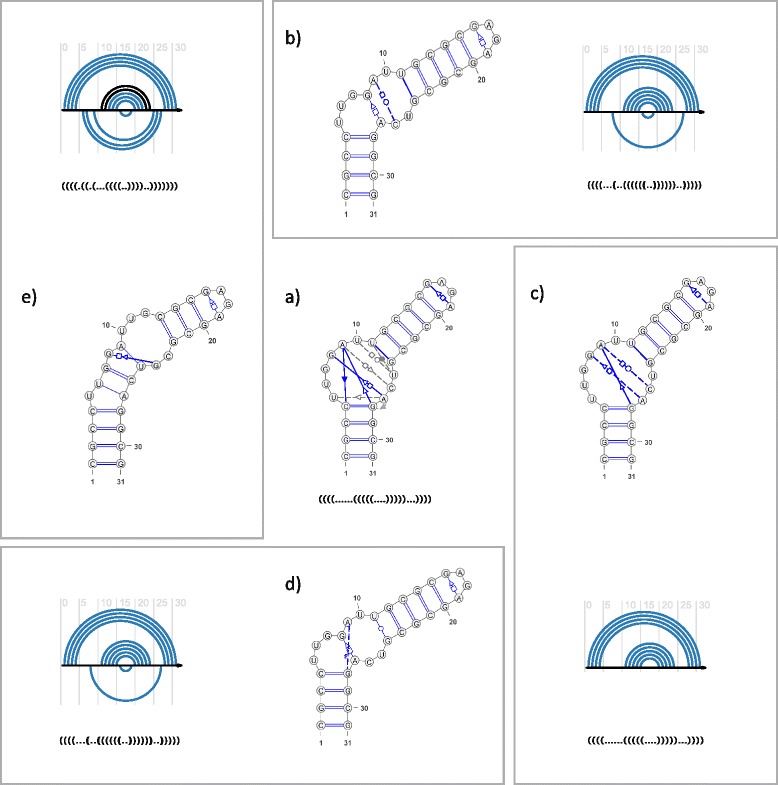
(**a**) The reference secondary structure of K-turn–GNRA construct with LW-annotated non-canonical base pairs, and its dot-bracket notation. Base pairs close to particular LW interaction, but not meeting strict criteria for membership are connected by gray dashed lines. (**b**-**e**) Secondary structures predicted by (**b**) RC/Rp-1, (**c**) RC/Rp-2, (**d**) RC/Rp-3, and (**e**) RNAwolf, and arc diagrams to display the results of comparing dot-bracket representations of particular predicted models with the reference structure

**Fig. 4:**
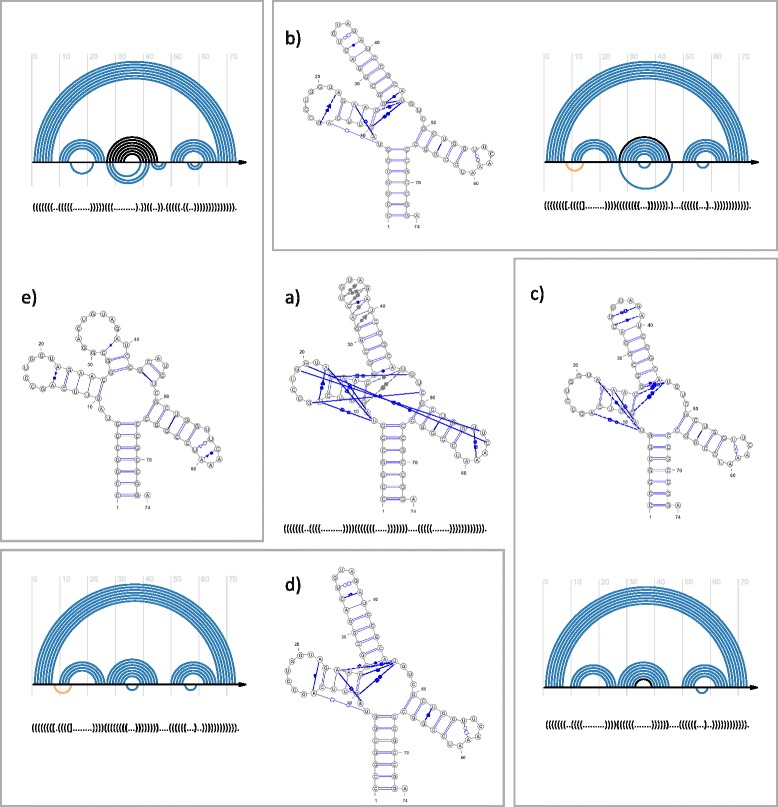
(**a**) The reference secondary structure of archaeal tyrosyl-tRNA with LW-annotated non-canonical base pairs, and its dot-bracket notation. Base pairs close to particular LW interaction, but not meeting strict criteria for membership are connected by gray dashed lines. **(b–e)** Secondary structures predicted by (**b**) RC/Rp-1, (**c**) RC/Rp-2, (**d**) RC/Rp-3, and (**e**) RNAwolf, and arc diagrams to display the results of comparing dot-bracket representations of particular predicted models with the reference structure. Orange arcs show pseudoknot interaction

To illustrate the results (Figs. 3 and 4) the secondary structure diagrams were prepared using VARNA [25], embodied into RNApdbee webserver. Additionally, arc diagrams were generated by R-CHIE software [26] from dot-bracket structure representations. Each arc diagram visualizes the result of comparing the predicted model to the reference secondary structure. Upper arcs represent predicted (blue) and unpredicted (black) base pairs that occur in the reference structure. Bottom arcs correspond to predicted base pairs that are not found in the reference structure. Thus, the blue upper arcs correspond to true positives, black upper arcs false negatives, and bottom arcs false positives. Dashed blue lines in structure images represent RC/Rp-predicted interactions mediated by one H-bond only. These interactions are not encoded in dot-bracket notation.

### A structure of K-turn–GNRA construct

The first example was constructed from a K-turn and GNRA loop. Its prediction aimed to check the ability of the RC/Rp pipeline and state-of-the-art methods to recognize the secondary structures of RNA modules. The K-turn sequence, r(CUUGGAUU).r(GUCAG) selected for the purpose of this experiment, came from the RNA component of the eukaryotic ribosome, deposited with IL_3U5F_051 identifier in RNA 3D Motif Atlas [27]. The corresponding crystallographic structure (PDB: 4 V88) facilitated a proper manual recognition of non-canonical interactions in the K-turn motif when constructing the reference model of the molecule. The GAGA loop was attached as the second component of the construct. Both components were connected by a sequence able to form a double-strand made of four canonical G–C pairs. Three additional three G–C base pairs were attached on the other side of K-turn motif. Thus, we obtained a construct with the following sequence: *5′-CGCCUUGGAUUGCGCGAGAGCGCGUCAGGCG-3′* and the secondary structure as shown in Fig. 3a.

Table 3 presents the results of comparing the reference structure to models predicted by all considered tools. The output from RC/Rp-1, RC/Rp-2, RC/Rp-3 and RNAwolf can be viewed in Fig. 3b-e. All tools were successful in predicting canonical interactions, while recognition of non-canonical ones revealed a visible difference in their performance. Most tools encountered some problems with the region containing the internal loop, which resulted in a low accuracy of K-turn interactions. MC-Fold and MC-Fold-DP did not recognize any non-canonical base pairs within the K-turn motif, while RNAwolf predicted one incorrect G–G pair there. The GAGA loop was mostly well predicted, although its non-canonical interaction (G16–A19) was not classified in the case of MC-Fold-DP. The RC/Rp pipeline was able to anticipate correctly most of the canonical base pairs and many non-canonical ones, although a few problems were encountered. RC/Rp-3 incorrectly recognized wobble U11–G24 as a non-canonical pair, since the 3DNA/DSSR procedure did not assign it to any Saenger class. RC/Rp-2 generated the best model (Fig. 3c). It found four non-canonical base pairs and correctly allocated three of them to LW families. Four non-canonical interactions were not found, three of them regarded as close to particular LW classes, and one being a strong non-canonical pair.

### A structure of archaeal tyrosyl-tRNA

This example molecule is a component of an archaeal tyrosyl-tRNA synthetase complexed with tRNA(Tyr) and L-tyrosine [23] (PDB: 1J1U). A structure of this complex was solved experimentally using X-ray crystallography with a resolution of 1.95 Å, and deposited in PDB [28]. Detailed structural information concerning just the RNA component, including non-canonical base pairs with classification, is available from NDB [29] (NDB: PR0092), while RNA STRAND [22] collects the basics of its secondary structure topology (RNA STRAND: PDB_00474). For the purpose of our experiment, the secondary structure of archaeal tyrosyl-tRNA taken from RNA STRAND was coupled with NDB-archived information about non-canonical interactions, thus constituting the reference structure (Fig. 4a).

Experimental results (Table 4) reveal that secondary models predicted by RNAwolf, MC-Fold-DP and MC-Fold significantly differ from the reference structure, especially where non-canonical interactions are concerned. MC-Fold-DP did not distinguish between canonical and non-canonical base pairs, thus giving the output structure quite distant from the reference one. RNAwolf and MC-Fold correctly predicted and classified one out of 18 non-canonical base pairs.

The RC/Rp pipeline correctly anticipated most of the canonical and non-canonical interactions (diagrams in Fig. 4b-d), including close to non-canonical ones (cf NDB [29]). As for canonical base pairs, only RC/Rp-1 gave a false positive (G25–U46). RC/Rp-3 provided zero false negatives and the number of true positives equal to the number of reference interactions. Additionally, all RC/Rp versions found one pseudoknot base pair and some distant interactions that, although not encoded in dot-bracket, existed in the reference structure. Due to the limitations of dot-bracket notation and incomplete encoding of the reference model in the RNA STRAND database, some correctly predicted interactions were counted as false positives (eg U55–A59 and U34–G38 pairs), while others were incorrectly classified as false negatives (eg G27–A45 and C33–A39 pairs from cis W-C/W-C family).

## Conclusions

We have demonstrated a novel approach for the automated assessment of extended RNA secondary structure from sequence or secondary structure. It is founded on the concept of annotating the extended RNA secondary structure on the tertiary coordinates, predicted in the preliminary step. We have shown its example implementation running RNAComposer and RNApdbee webservers in a computational sequence named the RC/Rp pipeline. The computational experiments performed on the entire set of sequences available from the RNA STRAND database, excluding those with modified residues, show the efficiency and superiority of our pipeline over the existing tools for assessing the extended secondary structure of RNA. It is particularly true as far as the accuracy of non-canonical base pair prediction is concerned. A detailed insight into two example structures of archaeal tyrosyl-tRNA and K-turn–GNRA construct also reveal the advantages of our approach over the other tools, especially in the case of non-canonical interactions. Even when faced with the K-turn motif that was difficult to identify [30], the RC/Rp pipeline was able to recognize many non-canonical interactions present there.

These promising results allow us to anticipate possible applications of the RC/Rp pipeline in different biological problems. The knowledge of extended secondary structure can accelerate an advancement of the 3D RNA module concept [31], and improve module identification and search within available structures [32]. We hope that the RC/Rp pipeline will be helpful in supporting new solutions to RNA motif discovery problems [33]. Indeed, in its first application to our previously-published data concerning the mechanism of spontaneous degradation of RNA molecules [34, 35], we found improved prediction accuracy of stable RNA degradants (data not shown).

Future plans include the development of a web server that will integrate both tools of the RC/Rp pipeline.

## Additional file

Additional file 1: Table S1. Accuracy of sequence-based prediction by RC/Rp pipeline applied for experimentally determined structures, assessed upon comparison of dot-bracket representations. **Table S2.** Accuracy of canonical secondary structure-based prediction by RC/Rp pipeline applied for experimentally determined structures, assessed upon comparison of dot-bracket representations. **Table S3.** Prediction accuracy for the reference set containing 1–200 nts long sequences and secondary structures from CompaRNA PDB benchmark. (PDF 78 kb)
